# Recycled polymer shot as sustainable additive for concrete: Mechanical, thermal, and environmental assessment

**DOI:** 10.1371/journal.pone.0342266

**Published:** 2026-02-09

**Authors:** Piotr Smarzewski, Marcin Małek, Janusz Kluczyński

**Affiliations:** 1 Institute of Civil Engineering, Department of Civil Engineering and Geodesy, Military University of Technology, Warsaw, Poland; 2 Military University of Technology, Faculty of Mechanical Engineering, Institute of Robots & Machine Design, Warsaw, Poland; Lovely Professional University, INDIA

## Abstract

The exponential growth of plastic production has led to a dramatic increase in plastic waste, creating significant environmental and public health challenges. Among the available disposal methods, recycling is widely regarded as the most environmentally and economically advantageous. The development of effective strategies for recycling plastic waste is therefore a critical area of research, particularly in the construction industry. Incorporating plastic waste into concrete not only minimizes landfill disposal, but also has the potential to improve the performance of the material. This study investigates the effect of recycled polymer shot, incorporated at levels of 5% and 10% by cement mass, on the physical, mechanical, and microstructural properties of concrete. Comprehensive tests were performed, including measurements of density, thermal conductivity, slump, slip resistance, compressive, flexural, and tensile strength, as well as elastic modulus. The results demonstrate that the use of polymer shot significantly improves key properties of concrete, with increases of up to 45% in flexural strength and 62% in tensile strength at the higher addition level. Image-based microstructural analysis further revealed modifications in porosity and fracture surface complexity, supporting the observed performance enhancements. Overall, the incorporation of recycled polymer shot in concrete provides a promising approach for valorizing plastic waste, improving concrete performance, and supporting sustainable construction and resource management strategies.

## 1. Introduction

In recent decades, a significant increase in global plastic production has been observed. In 2019, plastics production doubled compared to 2000, reaching 460 million tons [1]. This growth is primarily due to the advantageous properties of polymers, such as low density, high strength, ease of shaping, low thermal conductivity, long service life, and relatively low cost [2]. These features have made plastics indispensable in many sectors, including packaging, construction, automotive, electronics, and medicine, significantly improving functionality and efficiency in all industries [3].

However, the widespread use of plastics has led to a corresponding increase in waste generation, exceeding 300 million tons annually since 2019. Plastic waste presents major economic, environmental, and health challenges [4]. Currently, the main disposal methods include incineration, landfilling, and recycling [5]. Each of these approaches has certain drawbacks. Incineration leads to the emission of toxic compounds and greenhouse gases such as CO₂, NOₓ, and dioxins, which may pollute the air and threaten public health [6,7]. Landfilling, on the other hand, contributes to the long-term accumulation of non-biodegradable materials, with decomposition processes lasting up to 1000 years [8,9]. During degradation, various harmful substances can be released, leading to soil, water, and air contamination [10].

Recycling, although more environmentally friendly, is technologically complex. Polymer recycling includes stages such as collection, sorting, washing, shredding, and reprocessing [11]. During these steps, especially during mechanical recycling, polymers can be degraded due to thermal, chemical, or mechanical factors, which negatively affect their properties and usability [12]. Therefore, understanding the mechanisms of degradation and developing effective strategies to minimize performance loss in recycled plastics is a key area of current research [13,14].

An innovative and increasingly explored application of plastic waste is its use in concrete technology [15]. As one of the most widely consumed construction materials globally, concrete offers an opportunity for large-scale utilization of polymer waste. Incorporation of recycled polymers into concrete can improve not only its environmental footprint but also certain mechanical or durability-related properties [16–21]. This can also contribute to reducing cement usage, thus lowering CO_2_ emissions associated with clinker production [22].

Many studies have investigated the effects of the incorporation of polymer-based materials into concrete, primarily in the form of fibers or aggregates. Behzadian and Shahrajabian [23] added 10% PET waste and nanosilica to concrete, resulting in a 30% increase in compressive strength and a 27% improvement in tensile strength. Another study [24,25] tested PET, HDPE, and PP in various replacement ratios, confirming benefits such as improved abrasion resistance and acceptable shrinkage behavior. Mohammed et al. [26] found that up to 30% PVC waste can be used without compromising strength or workability. Research by Bambigboye et al. [27] demonstrated that partial replacement of coarse aggregates with PET enhanced workability and tensile strength, although compressive strength decreased beyond a certain content threshold.

The increasing generation of polymer waste has prompted research into effective recycling routes within construction materials. Incorporating recycled polymers into concrete allows for both material valorization and improved engineering performance, reducing the environmental footprint of the built environment.

Among various polymeric waste forms, polymer shot represents a specific class of recycled thermoplastic granules produced from post-industrial plastic residues. In contrast to polymer fibers or powders, the shot material has a nearly spherical or irregular geometry that may modify the microstructure and strength development mechanisms differently.

Therefore, this study aims to investigate the effect of recycled polymer shot addition at 5% and 10% by cement mass on the physical, mechanical, and environmental properties of concrete. The work is part of a broader effort to promote sustainable material design through the circular use of polymer waste in construction.

## 2. Materials

### 2.1. Cementitious materials and mix composition

The materials used in the study included Portland cement type CEM I 42.5R, following the standard PN-EN 197-1:2012 [28], tap water and a deflocculating agent – a third-generation polycarboxylate-based superplasticizer (3% by cement mass). Table 1 presents the chemical composition of the cement, determined according to the standard PN-EN 196-6:2019-01 [29]. The chemical composition of the cement sample was determined using X-ray fluorescence (XRF) analysis.

**Table 1:** Chemical composition of cement [29].

Compositions	SiO_2_	Al_2_O_3_	Fe_2_O_3_	CaO	MgO	SO_3_	Na_2_O	K_2_O	Cl
**Unit (vol.%)**	19.5	4.9	2.9	63.3	1.3	2.8	0.1	0.9	0.05

The XRF setup comprises an X-ray tube generating the primary X-ray beam (production system) equipped with primary collimators, crystals, secondary collimators, and detectors. The X-ray radiation generated by this system excites atoms in the sample, leading to the emission of radiation upon return to a stable state. The XRD system analyzes this emitted radiation. XRF measurements were performed using an ARL 9900 instrument (Thermo Fisher, Waltham, MA, USA), using monochromatic cobalt Kα1 radiation (wavelength = 1.788996 Å). Additionally, Table 2 presents the physical properties of the cement tested following the standard PN-EN 196-1:2016-07 [30].

**Table 2:** Physical properties of cement [30].

Specific surface area [m^2^/kg]	Specific gravity [kg/m^3^]	Compressive strength [MPa]	
		at 2 days	at 28 days
3840	3060	28.0	58.0

Table 3 presents the composition of 1 m^3^ of concrete mixture. As a filler, crushed polymer shot with particle sizes ranging from 0.1 to 2 mm was used. The polymer shot was introduced at 5% and 10% by mass of cement, replacing an equivalent volume of fine aggregate to maintain a constant total volume across all mixtures. The constant water-to-cement ratio (w/c = 0.30) was adopted in all mixtures.

**Table 3:** Concrete composition of 1 m^3.^

Compositions	Water [kg]	Aggregate [kg]	Cement [kg]	Polymer shot [kg]
**Reference**	205.86	1992.2	403.24	–
**M5**	205.86	1992.2	403.24	10.29
**M10**	205.86	1992.2	403.24	20.58

**Fig 1:**
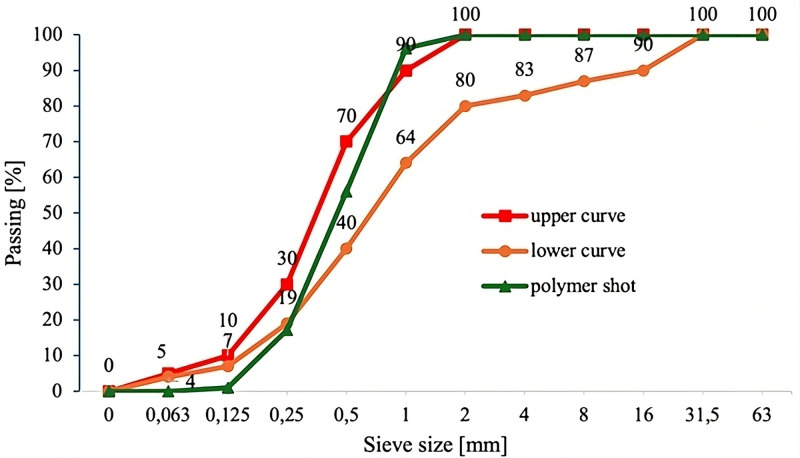
Gradation curve of polymer shot aggregate compared with standard lower and upper sand grading limits.

To verify compatibility with standard aggregate classification curves, a granulometric analysis was conducted. Fig 1 presents the particle size distribution of the polymer shot compared to the standard sand grading curves.

The distribution falls within the recommended range, indicating good packing potential for fine-aggregate replacement. In addition, the crushed particles featured irregular surface textures, attributed to mechanical or implosive fragmentation. This morphology may improve the mechanical interlock within the cementitious matrix.

### 2.2. Composition of polymer shot

The polymer shot was obtained from PP waste after multi-stage industrial processing including NIR-based sorting, washing, drying and fractionation and was characterized using PSD (laser diffraction), XRF (fine and coarse fractions), and SEM imaging.

The primary waste streams included food packaging (e.g., yogurt cups, margarine trays, lids), beverage containers, household chemical packaging, and production residues from the plastics industry. The materials were collected through selective municipal waste systems and directly from industrial facilities. The processing stages included:

(1) optical sorting using NIR sensors to separate PP from PET, PE-HD, PVC, and metals,
(2) hot alkaline washing to remove labels, adhesives and food residues,
(3) drying in forced-air tunnels or vertical centrifuges,
(4) mechanical shredding using knife mills operating at 300–1000 rpm with fixed screens (8–12 mm mesh),
(5) fractionation through air classifiers and RGB camera sorting.

It should be noted that mechanical recycling processes (e.g., shredding, grinding and repeated handling) may induce polymer degradation, including chain scission and thermo-oxidative effects, which can alter molecular weight and melt flow behavior compared to virgin PP. In the present study, the recycled polymer shot was used as a solid particulate inclusion in a cementitious matrix. Therefore, the concrete performance is governed primarily by particle geometry, dispersion and the polymer–cement interfacial transition zone. Nevertheless, potential processing-related degradation may contribute to batch-to-batch variability and should be further quantified in future studies using polymer-specific characterization (e.g., DSC, FTIR or MFI), as discussed in Section 4.8.

**Fig 2:**
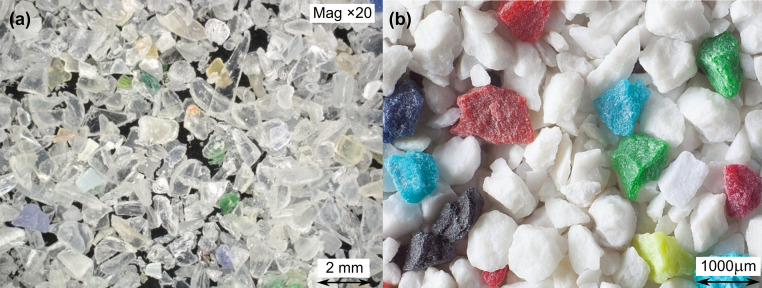
Optical images of polymer shot aggregate: (a) coarse fraction, × 20 magnification; (b) fine-to-medium mixed fraction, × 20 magnification.

This technology yielded PP flakes with irregular geometry and relatively consistent particle size, as seen in Fig 2.

The polymer shot consisted of two types of particles (fine and coarse) with sharp, angular shapes and irregular contours. These properties stem from mechanical fragmentation rather than thermoplastic molding, ensuring surface roughness favorable for cement adhesion.

The physical properties of the polymer shot are presented in Table 4. The hardness (Mohs scale), specific gravity and bulk density values confirm its lightweight and moderately hard character, suitable for use as a concrete filler or lightweight aggregate.

**Table 4:** Physical properties of polymer shot.

Property	Hardness (Mohs scale)	Specific gravity [g/cm³]	Bulk density [kg/m³]
Polymer shot	3.5 ± 0.183	1.5 ± 0.153	907.4 ± 3.194

**Fig 3:**
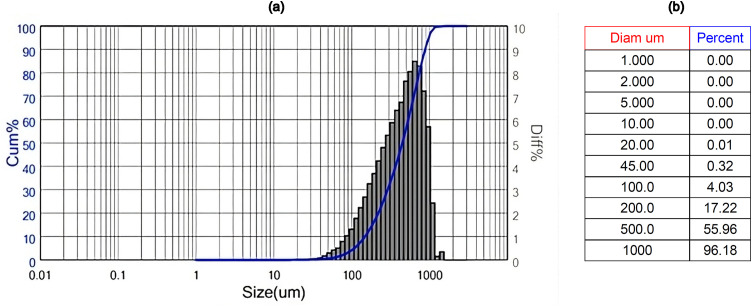
Particle size distribution of the polymer shot obtained by laser diffraction analysis: (a) histogram with cumulative curve; (b) selected particle diameters and corresponding cumulative passing percentages.

Subsequently, the particle size distribution was assessed using laser diffraction analysis and detailed data are summarized in Fig 3. The results show a broad particle distribution with D90 > 870 µm, confirming the dominance of coarse flakes.

The chemical composition of both fine and coarse fractions of the polymer shot was determined by X-ray fluorescence spectroscopy (XRF), and the results are shown in Table 5. The high carbon and oxygen contents confirm the organic nature of the recycled polymer material.

**Table 5:** Combined chemical composition of polymer shot, including both fine and coarse fractions (XRF analysis).

	Fine fraction		Coarse fraction	
Element	Atomic [%]	Weight [%]	Atomic [%]	Weight [%]
**Carbon**	75.56	27.44	70.47	64.01
**Oxygen**	66.50	33.50	29.41	35.58
**Aluminum**	−	−	0.09	0.18
**Niobium**	−	−	0.03	0.21
**Zirconium**	−	−	0.00	0.01

**Fig 4:**
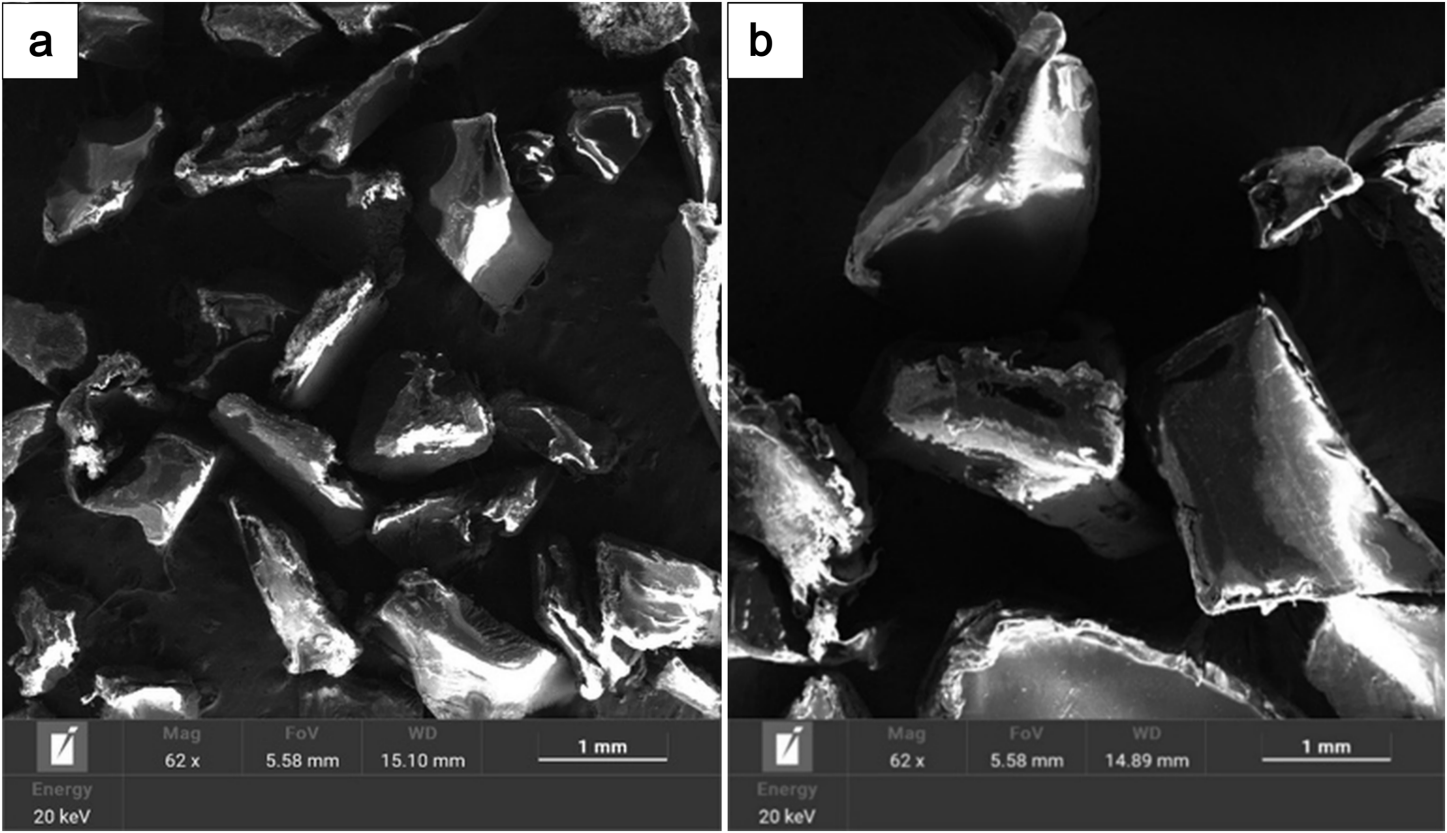
SEM microstructure (×62 magnification) of polymer shot: (a) fine particles; (b) coarse particles.

Scanning electron microscopy (SEM) was also employed to evaluate the surface morphology. As seen in Fig 4, the polymer shot exhibits an angular surface texture with occasional surface pitting, which may influence bonding performance in cementitious matrices.

The properties mentioned above make the polymer shot a promising candidate for lightweight construction materials, surface treatments, or as an additive in polymer-modified concrete where control over density and hardness is critical.

### 2.3. Production of the cementitious blend

The prepared cementitious blend was supplemented with polymer shot at a ratio of 5% and 10% by mass of cement, to investigate the effect of polymer shot content on the properties of the cementitious blend. These dosages were selected based on preliminary mixing trials and practical considerations. The 5% level represents a moderate addition, whereas 10% was adopted as an upper bound that remained feasible at a constant w/c ratio and enabled evaluation of a clear dose–response relationship and potential trade-offs in fresh and hardened properties. A constant water-to-cement ratio (w/c = 0.3) maintained constant for all blends, and the admixture content was 3% by mass of cement, following [31,32]. The polymer shot was added to the cementitious blend after the preliminary mixing of cement, sand, and water for 2 minutes, and the total mixing time did not exceed 5 minutes. To promote uniform dispersion, the polymer shot was introduced gradually after the initial wetting of the cementitious matrix, and mixing was continued for the remaining time to minimize the risk of particle clustering and formation of polymer-rich zones, including at the 10% content. Samples were prepared under controlled laboratory conditions at 21°C and 50% relative humidity. Subsequently, the samples were stored in water according to PN-EN 12390–2:2019 [33].

## 3. Methodology

### 3.1. Physical properties testing

The thermal conductivity coefficient was characterized using an ISOMET 2114 analyzer (Applied Precision Ltd. Bratislava, Slovakia), which is equipped with a resistive heater for the precise measurement of the temperature response to heat flow impulses.

The density of the cured samples was determined according to EN 12390–7:2019-08 [34], calculated as the ratio of the mass of the sample to its volume. Measurements were performed using a VIBRA analytical balance (Kraków, Poland) and electronic calipers (TOYA, Wrocław, Poland).

### 3.2. Slump test

**Fig 5:**
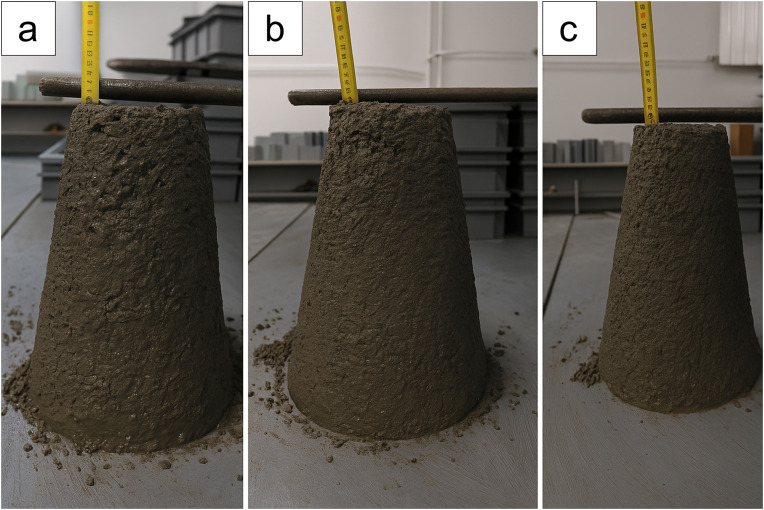
Slump test of fresh concrete mixtures: (a) reference sample, (b) sample containing 5% polymer shot (by mass of cement), and (c) sample containing 10% polymer shot (by mass of cement).

The workability of the fresh concrete mixture was evaluated by the slump test in accordance with PN-EN 12350−2 [35] using equipment supplied by Merazet (Poznań, Poland) (Fig 5).

### 3.3. Mechanical properties

Mechanical properties, including flexural strength, compressive strength, and splitting tensile strength, were evaluated using a Zwick testing machine (Ulm, Germany; force range: 0–5000 kN).

**Fig 6:**
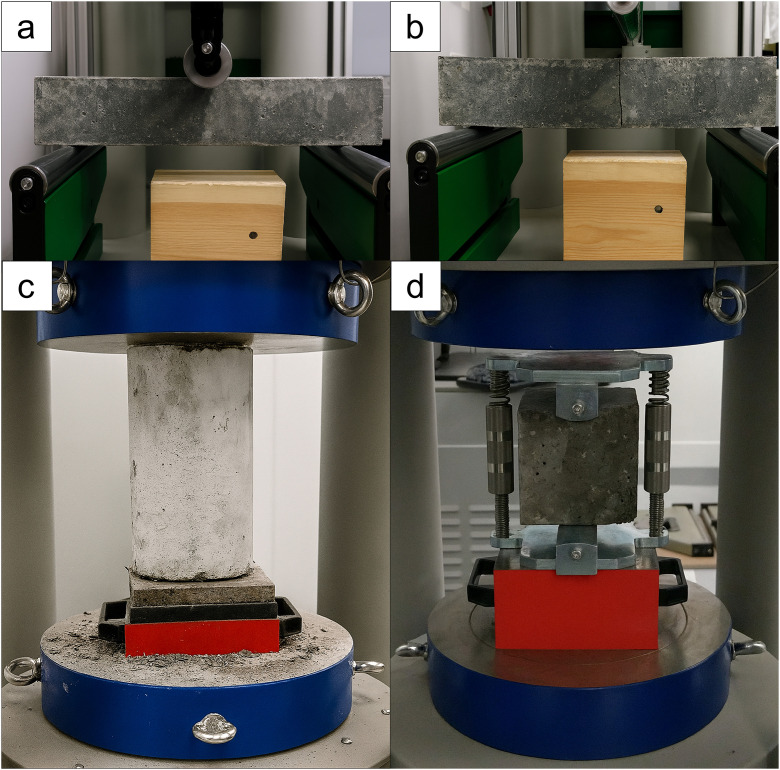
Mechanical tests a) before the flexural strength test, b) after the flexural strength test, c) compressive strength test, and d) splitting tensile strength test.

Flexural strength measured by the three-point bending method on prisms (40 mm × 40 mm × 160 mm) with standard support spacing, allowing horizontal roller movement, in accordance with EN 12390–5:2019-08 [36] (Fig 6a, b). Compressive strength was determined in accordance with EN 12390–3:2019-07 [37] on cylindrical specimens (150 mm × 300 mm) (Fig 6c). The tensile splitting strength was measured according to EN 12390–6:2011 [38] on cubic specimens (100 mm × 100 mm × 100 mm) (Fig 6d). The modulus of elasticity was determined according to EN 12390–13:2014-02 [39] using cylindrical specimens (150 mm diameter, 300 mm height). Two electrical resistance strain gauges (100 mm gauge length) were mounted on opposite sides at mid-height. Axial and transverse deformations were recorded using Epsilon strain gauges (Epsilon, Jackson, USA). The surfaces of the specimens were ground to ensure parallelism before testing. Each specimen was subjected to three loading and unloading cycles within the specified stress ranges. The modulus of elasticity was calculated on the basis of the recorded deformations and the gauge length of the devices.

For each mixture, mechanical properties were determined using n = 5 specimens for compressive strength, n = 5 specimens for splitting tensile strength, and n = 5 specimens for flexural strength. Physical properties were measured using n = 5 specimens per mixture. The full raw dataset is provided in S2 File.

### 3.4. Slip resistance value (SRV)

The slip resistance value (SRV) was measured using a British Pendulum Tester (WESSEX, Aldershot, United Kingdom), according to EN 14231 [40] and CEN/TS 16165, Annex C [41]. The device used a type 57 rubber slider CEN (hardness: 55–61 IRHD, width: 76.2 mm, length: 126 mm). Calibration was performed with reference substrates (glass, tiles and polishing paper). Measurements were conducted under dry and wet conditions (with distilled water) on two samples, with five measurements for each condition.

### 3.5. Image analysis of microstructure and pore size distribution

The microstructural analysis of the fracture surfaces was performed on representative optical micrographs acquired at 50 × magnification using a Keyence VHX-7100 digital microscope (Keyence Corporation, Osaka, Japan) with a calibrated scale bar (100 μm or 50 μm, as indicated on each image). The images were saved in high-resolution format (minimum 300 dpi) and subjected to quantitative analysis using the open-source Python libraries scikit-image and numpy.

Before analysis, the color micrographs were converted to 8-bit grayscale. To separate the pore space (dark areas) from the solid matrix, the global thresholding method of Otsu [42] was applied. This algorithm automatically determines the optimal threshold value that minimizes intra-class intensity variance, allowing for robust and objective binarization of the images without subjective parameter selection.

For each image, the threshold value determined by the Otsu algorithm was applied, resulting in binary images where the pores were represented as black pixels (value 0) and the solid phase as white pixels (value 1). The resulting binary images retained the original field of view and scale bar.

The surface porosity (2D area fraction of pores) was calculated as the ratio of the number of black (pore) pixels to the total number of pixels in the field of view, expressed as a percentage:


$$\text{Porosity (\textbackslash{}\%)}=\frac{N_{\text{pore pixels}}}{N_{\text{total pixels}}}\times 100$$ (1)

To further characterize the microstructure, we quantified the pore size distribution for each binarized image. Individual pores were identified as connected black regions using the label function of the scikit-image library. To reduce noise and exclude very small artifacts, only pores with an area ≥ 15 pixels were included in the pore-size analysis. The equivalent pore diameter (*d*_eq_) for each pore was calculated as the diameter of a circle with the same area as the measured pore


$$d_{\text{eq}}=2\sqrt{\frac{A_{\text{pore}}}{\pi }}$$ (2)

where *A*_pore_ is the area of the pore in pixels converted to micrometers squared on the basis of the image scale. The distribution of the equivalent pore diameters is then presented in Section 4.5. All image processing and analysis steps were performed using Python 3.11, scikit-image v0.22.0, numpy v1.26.4, and matplotlib v3.7.1. The same workflow was applied to all images to ensure comparability between samples. The field of view for each micrograph was approximately 1,120 × 840 μm, and the minimum detectable pore size was limited by image resolution (1 pixel ≈ 1.5 μm, based on the calibration bar in the images).

### 3.6. Fractal dimension and texture roughness indicators

Quantitative assessment of fracture surface complexity and texture roughness was performed on binarized optical micrographs using three complementary indicators: fractal dimension (*D*), mean gradient (*G*), and image entropy (*H*).

The fractal dimension (*D*) of each fracture surface was determined using the box-counting algorithm applied to binary images [43]. The image was covered with a series of square boxes of size *ε*, and the number of boxes *N*(*ε*) containing part of the pore–matrix interface was counted. The size of the box was varied on a range of scales and the fractal dimension was estimated as the slope of the regression line fitted to the log–log plot of *N*(*ε*) vs. 1/*ε*


$$D=-\underset{\varepsilon \to 0}{lim}\frac{logN\left(\varepsilon \right)}{log\varepsilon }$$ (3)

A higher fractal dimension indicates a more complex, irregular surface morphology.

The mean gradient (*G*) quantifies the average change in grayscale intensity between adjacent pixels, providing a measure of surface roughness and edge density [44]:


$$G=\frac{\text{1}}{N}\sum_{i=1}^{N}\left|I_{i+1}-I_{i}\right|$$ (4)

where *I*_*i*_ is the intensity of the *i*-th pixel and *N* is the total number of pixel pairs in the image.

The mean gradient was calculated as above and the result was normalized to the range [0, 1] by dividing by 255.

The image entropy (*H*) was calculated from the normalized histogram of grayscale values, quantifying the randomness and complexity of the surface texture [45]:


$$H=-\sum_{i}p_{i}{log}_{\text{2}}p_{i}$$ (5)

where *p*_*i*_ is the probability of the *i*-th intensity value. Higher entropy values reflect greater heterogeneity and disorder in texture.

All calculations were performed using custom Python scripts (Python 3.11, scikit-image v0.22.0, numpy v1.26.4).

Ethics statement: not applicable. No human participants or animals were involved in this study.

## 4. Results and discussion

### 4.1. Fresh properties (slump test)

**Fig 7:**
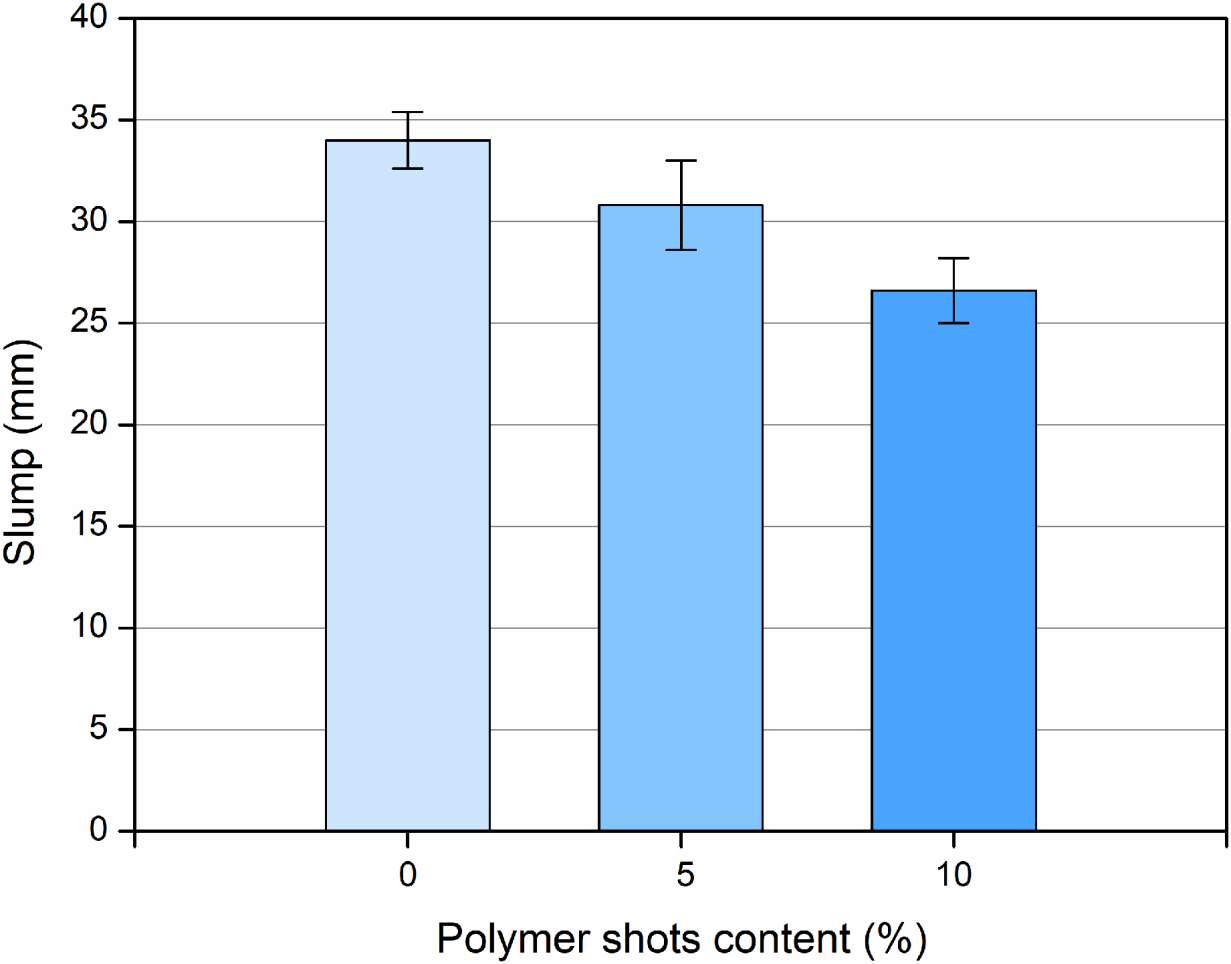
Slump of concrete mixes with varying polymer shot content.

The assessment of the fresh properties of mortar mixtures was conducted based on the slump test, the results of which are presented in Fig 7.

All presented results are the mean values from five measurements of samples for each mixture. During the analysis of all mortar compositions, no cases of segregation or bleeding were observed during the mixing and pouring process.

All tested mixtures were classified into the S1 slump class. For the reference samples, the slump height was 34.0 ± 1.4 mm. For mixtures containing 5% and 10% additions of polymer shot, respectively, a decrease in the slump height was observed. For the mixture with a 5% addition of polymer shot, the slump decreased by approximately 9.5%, reaching a value of 30.8 ± 2.2 mm, while for the mixture with a 10% addition, the slump decreased by approximately 22%, reaching a value of 26.6 ± 1.6 mm.

Although these reductions were moderate, they confirm the tendency of polymer shot to reduce workability due to the hydrophobic nature and irregular surface texture of the particles. The relatively low slump values were intentionally adopted to ensure a dense and homogeneous microstructure suitable for structural or prefabricated applications. This approach aligns with mix design principles for low-porosity concretes. However, for practical applications requiring higher consistency, the workability loss at 10% polymer shot content may be relevant and can be mitigated through mix design optimization. Potential strategies include adjusting the type and dosage of superplasticizer, refining the particle grading and paste volume, and conditioning the polymer shot (e.g., pre-wetting or mild surface treatment) to improve dispersion and reduce water demand, while maintaining the targeted hardened-state performance.

Researchers observed significantly greater decreases when polymer fibers were used as an additive. After adding 0.5%, 1%, and 1.5% by weight of fibers, the slump reduction increased by 22.9%, 57.1%, and 95.7%, respectively, compared to regular concrete. Similar high reductions were observed by other authors [46,47], who obtained slump reduction values close to 88%.

Thus, the use of polymer shot, unlike polymer fibers, provides a more moderate effect on the rheological properties of the mixture, maintaining practical workability.

### 4.2. Physical properties

The results of the physical properties of the tested samples (bulk density and thermal conductivity coefficient) are presented in Table 6.

**Table 6:** The results of the physical properties of the tested samples.

Identification of the tested sample	Density [kg/m^3^]	Thermal conductivity coefficient [W/m ⋅ K]
Ref. - Reference sample	2166 ± 30	1.6376 ± 0.0350
M5 - Addition of 5% by mass of cement of polymer shot	2213 ± 26	0.9807 ± 0.0158
M10 - Addition of 10% by mass of cement of polymer shot	2228 ± 13	0.9150 ± 0.0084

Note: Unless stated otherwise, all values in Tables 6–10 are reported as mean ± SD based on five specimens per mixture (n = 5). Raw data are provided in S2 File.

The addition of polymer shot resulted in a slight increase in the density of the samples. The density of the samples increased by 2% and 3%, respectively, with increasing addition of polymer shot. Although the polymer has a lower intrinsic density than the mineral aggregate, the overall increase can be attributed to improved particle packing and reduced entrapped air, promoted by the angular geometry of the polymer shot and its ability to fill microvoids between sand grains.

However, the addition of polymer shot significantly reduced the thermal conductivity of the samples by approximately 40% with a 5% shot addition and 44% with a 10% shot addition, compared to the reference sample. This effect directly improves the insulation performance of the produced concretes, suggesting their suitability for elements requiring both mechanical strength and low heat transfer.

Researchers [48] who utilized polymer fibers in concrete production also did not observe significant changes in the density of the tested samples. However, different results were obtained by Al-Hadithi and Hilal [49], who demonstrated that increasing the volumetric content of polymer fibers from 0% to 2% resulted in a decrease in density from 2340 kg/m^3^ to 2235 kg/m^3^. Similarly, other studies [50,51] reported a decrease in density with increasing polymer content.

In contrast, the slight densification observed here may indicate better internal compaction and an optimal vibration time during casting.

The reduction in thermal conductivity is consistent with previous studies [52,53] and confirms the potential of polymer shot for use in energy-efficient concrete components.

### 4.3. Mechanical properties

The mechanical properties of the specimens are presented in Table 7.

**Table 7:** Mechanical properties of the samples tested.

Identification of sample	Flexural strength [MPa]	Compressive strength [MPa]	Tensile strength [MPa]	Elastic modulus [GPa]
Ref.	5.95 ± 0.048	42.91 ± 0.718	2.53 ± 0.027	30.70 ± 0.470
M5	8.00 ± 0.075	45.36 ± 0.195	3.53 ± 0.040	31.48 ± 0.510
M10	8.61 ± 0.090	49.49 ± 0.569	4.10 ± 0.025	32.02 ± 0.341

With an increase in polymer shot content, the mechanical strength of the specimens improved in all test categories. The flexural strength increased by 34% and 45% for the 5% and 10% additions, respectively. Compressive strength increased by 6% and 15%, while tensile strength increased by 40% and 62%. The modulus of elasticity also showed a moderate increase of 2.5% and 4%.

These results indicate that polymer shot inclusions can enhance the load-carrying capacity and stiffness of the concrete matrix. The observed improvement in tensile and flexural strength can be attributed to microcrack-bridging effects and energy dissipation induced by the polymer inclusions, which help delay crack propagation and increase post-peak ductility.

In addition, the irregular geometry of the polymer particles and their mechanical interlock with the cement paste likely contributed to improved bonding, providing resistance to localized stress concentrations.

**Fig 8:**
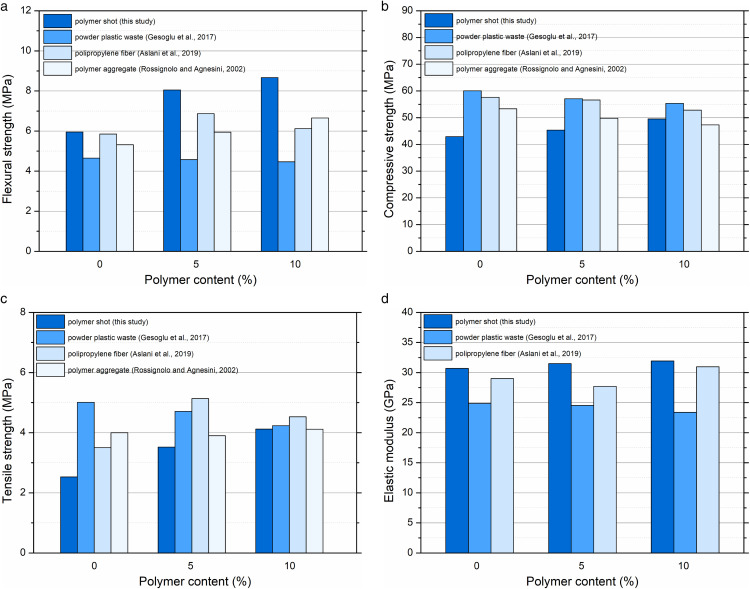
Effect of different polymer additives on the mechanical properties of concrete at 0%, 5%, and 10% content: (a) flexural strength results, (b) compressive strength results, (c) splitting tensile strength results, and (d) elastic modulus results.

The obtained results from the mechanical tests were compared with other available studies [51,52,54,55], where similar improvements were noted for polymer or hybrid fiber-reinforced concretes (Fig 8). For example, Gesoglu et al. [54] reported a 9–19% increase in compressive strength with the inclusion of 4–6 kg/m^3^ of polymer fibers, although the modulus of elasticity remained almost unchanged due to the low fiber volume fraction (< 0.7%). Moreover, tensile strength increased by 29–42%, consistent with the trends observed in this study.

Conversely, some authors [52,56] reported decreases in compressive strength when excessive polymer contents were used, particularly in self-compacting or high-workability mixtures. The balanced enhancement observed in the present work suggests that polymer shot, with its irregular shape and moderate dosage, is more effective than long polymer fibers or smooth pellets.

Overall, the introduction of polymer shot led to a synergistic improvement in both mechanical and physical parameters without compromising workability. The results demonstrate that this type of recycled polymer material can be successfully applied in sustainable concrete production while maintaining or improving strength characteristics.

### 4.4. Slip resistance

The slip resistance tests, as summarized in Table 8, revealed a marked increase in the slip resistance value (SRV) of concrete with increasing polymer shot content. The SRV improved from 60.09 ± 0.85 for the reference sample to 76.67 ± 1.00 and 85.47 ± 0.75 for concretes containing 5% and 10% polymer shot, respectively. This translates to a substantial enhancement of 27.2% and 42.2% compared to the unmodified concrete.

**Table 8:** Values of slip resistance of samples.

Identification of sample	Slip resistance value
Ref.	60.09 ± 0.852
M5	76.67 ± 1.000
M10	85.47 ± 0.748

This significant improvement in slip resistance can be attributed to changes in both the micro- and macrotexture of the concrete surface induced by the addition of polymer shot. The presence of polymer shot particles likely introduces additional surface irregularities and microasperities, contributing to increased friction at the interface between the surface and the sliding object. Furthermore, uniform dispersion of the shot within the matrix can help prevent surface polishing and reduce the tendency for smooth, slippery surfaces to develop during finishing or use.

From a practical point of view, improved slip resistance is a highly desirable property in many concrete applications. Surfaces with improved anti-slip performance are essential in areas with high pedestrian or vehicular traffic, such as sidewalks, ramps, driveways, parking garages, and industrial floors. In such settings, reducing the risk of slips and falls is a critical safety concern, and materials that can provide long-lasting, reliable slip resistance are particularly valuable.

The present results indicate that incorporating polymer shot into concrete mixtures not only improves mechanical and thermal performance but also offers a significant functional advantage in terms of safety. This positions polymer shot-modified concrete as an attractive solution for environments where slip resistance is mandated by regulations or strongly recommended by best practice guidelines (e.g., public spaces, transport infrastructure, healthcare or educational facilities).

It should also be noted that, unlike some additives, such as nano-CaCO_3_ or microfibers, which can decrease slip resistance at low doses and require careful optimization of content [57], the use of polymer shot appears to produce consistent and substantial improvements even at moderate replacement levels. This ease of implementation further enhances its appeal for industry adoption. Beyond the direct numerical improvements, these findings carry important practical implications for pavement engineering. The significant improvement in slip resistance observed in polymer shot-modified mixtures has direct implications for pavement safety, as higher SRV values correspond to reduced skidding potential. Furthermore, the reduction in thermal conductivity suggests potential applications in pavement layers exposed to extreme thermal gradients, where improved insulation can mitigate temperature-induced cracking. These findings highlight the potential of polymer shot to be adopted in pavement engineering, particularly for precast elements, walkways, and road surface layers requiring enhanced skid resistance.

In summary, the substantial increase in slip resistance achieved by adding a polymer shot broadens the potential applications of this modified concrete, offering both enhanced safety and improved durability, while also contributing to the sustainable management of polymer waste.

### 4.5. Microstructural analysis

**Fig 9:**
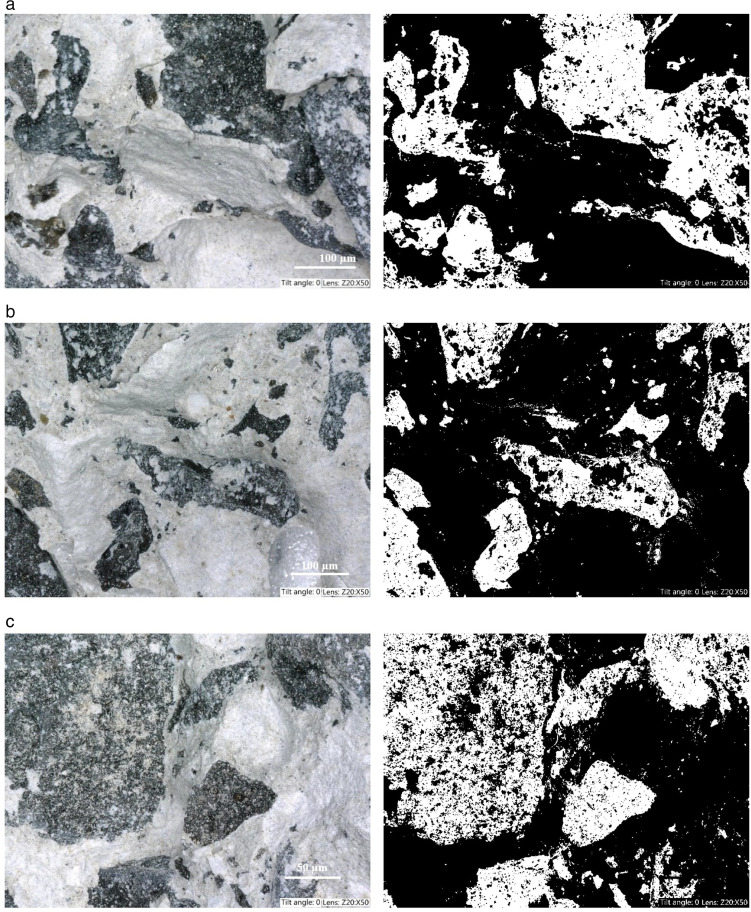
Representative optical micrographs (left) and binarized pore structure images (right) of the fracture surfaces: (a) reference sample, (b) sample with 5% polymer shot addition, (c) sample with 10% polymer shot addition.

Representative optical micrographs and the corresponding binarized pore structure images for the reference sample, as well as samples with 5% and 10% polymer shot addition, are presented in Fig 9. The images illustrate the fracture surface morphology and allow for a comparative analysis of the pore area fraction between mixtures.

Quantitative image analysis revealed that the reference sample exhibited a surface porosity (area fraction of pores) of 33.7%. The incorporation of 5% polymer shot resulted in a significant reduction in surface porosity to 22.6%. Conversely, increasing the polymer shot content to 10% led to a marked increase in the observed porosity, reaching 37.5%. These findings indicate that a moderate addition of polymer shot may contribute to matrix densification and pore structure refinement, while excessive shot content can potentially increase the overall interfacial transition zone (ITZ) area and promote entrapped air and microvoid formation, resulting in a higher pore volume and/or larger pore sizes.

At first glance, the simultaneous increase in porosity descriptors including the higher apparent porosity and surface disorder at 10% polymer shot content, and the continued increase in strength may seem contradictory. However, the porosity metrics derived from 2D optical image analysis represent local cross-sectional void features and can be strongly influenced by a limited number of isolated macrovoids. Such macrovoids may markedly increase the measured porosity in 2D images, while their effect on compressive strength can remain limited if they are sparse and do not form a connected pore network. In parallel, the microstructure at higher polymer content may exhibit refinement of the smaller pore population and densification of the load-bearing cementitious matrix, which governs the overall mechanical response.

Furthermore, the increased polymer volume fraction modifies the matrix–inclusion interface area and may contribute to crack-arrest mechanisms by locally deflecting or bridging microcracks, particularly under tensile and flexural loading, which can translate into improved mechanical performance even when image-based porosity increases. Therefore, the observed trends suggest that strength is controlled primarily by the continuity and quality of the cementitious skeleton and the refined microstructure at the microscale, whereas the increase in apparent porosity at 10% content is associated with localized macrovoids and higher surface heterogeneity rather than a uniform degradation of the matrix.

The observed changes in the pore structure are consistent with the trends in mechanical and thermal properties described in the previous sections, further confirming the significant influence of the polymer shot content on the microstructural characteristics of the composite.

The results summarized in Table 9 indicate that the mixture containing 5% polymer shot exhibits the lowest median pore diameter and a generally refined pore structure, as reflected by reduced characteristic pore sizes (median and upper percentiles). This suggests a partial densification of the microstructure. In contrast, the mixture with 10% polymer shot shows a higher total number of pores and a wider dispersion, characterized by relatively low percentile values but the occurrence of occasional very large pores (high maximum value). This pattern may indicate the formation of localized macrovoids, potentially related to entrapped air and the increased polymer–cement interfacial transition zone (ITZ) area at higher shot content, which could negatively influence durability despite the overall refinement of the smaller pore population.

**Table 9:** Pore size statistics for analyzed mixtures (equivalent diameter, μm).

Statistic	Reference	5% Polymer shot	10% Polymer shot
Number of pores	244	344	519
Median	13.00	11.42	10.30
75th percentile	22.49	21.26	15.65
90th percentile	52.27	44.58	25.17
Maximum	1289.74	692.73	1319.85

Analysis of surface complexity and texture roughness revealed distinct differences between the tested samples (Table 11).

**Table 10:** Fractal dimension and texture roughness indicators for analyzed fracture surfaces.

Sample	Fractal dimension (*D*)	Mean gradient (*G*) [0–1]	Entropy (*H*)
Reference	1.607	0.0121	7.48
5% polymer shot	1.604	0.0146	7.11
10% polymer shot	1.730	0.0196	7.51

**Table 11:** ANOVA and Tukey’s HSD results for key properties.

Property	ANOVA (p-value)	Tukey’s HSD (p < 0.05)
Density [kg/m³]	< 0.001	Ref ≠ M5, Ref ≠ M10, M5 ≠ M10
Thermal conductivity [W/m·K]	< 0.001	Ref ≠ M5, Ref ≠ M10, M5 ≠ M10
Flexural strength [MPa]	< 0.001	Ref ≠ M5, Ref ≠ M10, M5 ≠ M10
Compressive strength [MPa]	< 0.001	Ref ≠ M5, Ref ≠ M10, M5 ≠ M10
Tensile strength [MPa]	< 0.001	Ref ≠ M5, Ref ≠ M10, M5 ≠ M10
Elastic modulus [GPa]	< 0.001	Ref ≠ M5, Ref ≠ M10, M5 ≠ M10
Slip resistance (SRV)	< 0.001	Ref ≠ M5, Ref ≠ M10, M5 ≠ M10

Note: ANOVA assumptions were verified using Shapiro–Wilk and Levene tests (p > 0.05 in all cases).

The reference mixture exhibited a fractal dimension of *D* = 1.607, accompanied by a moderate mean gradient (*G* = 0.012) and entropy (*H* = 7.48), indicating an intermediate level of fracture surface complexity and texture heterogeneity.

The incorporation of 5% polymer shot slightly reduced the fractal dimension (*D* = 1.604), suggesting a marginally smoother and less complex fracture morphology. At the same time, the mean gradient increased (*G* = 0.015), which may reflect the formation of finer edge features and an enhanced density of micro-scale transitions in grayscale intensity. Notably, the entropy value (*H* = 7.11) was the lowest among all mixtures, indicating a more ordered and homogeneous texture. In contrast, increasing the polymer shot content to 10% led to the highest fractal dimension (*D* = 1.730) and the highest mean gradient (*G* = 0.020), indicating a more irregular and rougher fracture surface. The entropy was also slightly higher (*H* = 7.51) than in the reference sample, implying increased heterogeneity and disorder in texture.

Overall, these indicators suggest that a moderate polymer shot content (5%) promotes microstructural uniformity, whereas excessive addition (10%) increases fracture surface complexity and texture roughness. This observation is consistent with the higher measured surface porosity of the 10% mixture and the presence of occasional large voids identified in the pore-size analysis.

### 4.6. Statistical analysis

Statistical analyses were performed using the raw results from five specimens per mechanical property and per mixture (n = 5), as provided in S2 File. All underlying raw values used to calculate means, standard deviations, and to perform ANOVA are freely available in the Supporting Information (S2 File).

The statistical evaluation of the experimental results confirmed that the addition of a polymer shot significantly improved the performance of concrete in a dose-dependent manner. Table 11 summarizes the results of the ANOVA and Tukey’s HSD tests. Prior to ANOVA, the assumptions of normality and homogeneity of variances were verified. Normality of residuals was evaluated using the Shapiro–Wilk test, and equality of variances was assessed using Levene’s test (α = 0.05). Since ANOVA assumptions were satisfied (p > 0.05), standard one-way ANOVA was applied. Welch’s ANOVA was additionally used as a robustness check and yielded consistent conclusions. One-way analysis of variance (ANOVA) revealed statistically significant differences (p < 0.001) between the reference mixture and both modified compositions (M5 and M10) for all parameters tested. Post-hoc Tukey’s HSD tests further demonstrated that every pairwise comparison (including Ref vs. M5, Ref vs. M10, and M5 vs. M10) showed significant distinctions (p < 0.05), indicating progressive improvement with increasing polymer shot content.

The most notable improvement was observed in mechanical properties, where the flexural strength increased by 34% and 45% for M5 and M10 respectively, while tensile strength exhibited even greater improvements of 40% and 62%. Compressive strength showed more modest but still significant gains of 6% and 15% for the 5% and 10% formulations. These mechanical improvements were accompanied by a substantial 44% reduction in thermal conductivity for the M10 mixture, suggesting excellent potential for thermal insulation applications.

Equally important were the modifications of the surface properties, with the slip resistance values (SRV) increasing progressively by 27% and 42% for M5 and M10. The consistent statistical significance across all property categories and mixture comparisons (p < 0.05) provides robust evidence that polymer shot incorporation yields measurable and predictable enhancements in concrete performance. These findings strongly support the feasibility of using recycled polymer shot as a functional additive that simultaneously improves multiple concrete characteristics while contributing to sustainable construction practices.

The clear dose-response relationship observed across all tested parameters suggests that the optimal polymer shot content can be selected based on specific application requirements, with higher percentages (up to 10%) providing the most substantial performance benefits without compromising other material properties.

### 4.7. Sustainability and practical relevance

The use of recycled polymer shot in concrete directly contributes to the principles of circular economy and sustainable construction by replacing a portion of natural fine aggregate with a recovered material derived from post-consumer and industrial plastic waste.

Each cubic meter of concrete containing 10% polymer shot (by cement mass) utilizes approximately 40 kg of recycled polypropylene (PP) waste, preventing its disposal in landfills and reducing the need for virgin sand extraction.

In addition to the environmental benefits, the improved physical and mechanical performance of the polymer shot-modified concrete enhances its long-term durability and functional properties. The reduced thermal conductivity indicates potential for energy-efficient building envelopes, while the increased slip resistance suggests suitability for walkways, ramps, and pavement blocks exposed to wet or polished surfaces.

A simplified environmental assessment was performed to estimate the embodied energy and CO_2_ reduction potential, based on typical literature values for raw material production and transport (Table 12). The results are intended to illustrate the order of magnitude rather than provide a full LCA.

**Table 12:** Simplified environmental indicators for polymer shot-modified concrete.

Parameter	Reference concrete	M5 (5% polymer shot)	M10 (10% polymer shot)
CO_2_ emission reduction (kg/m^3^)	—	0.25	0.40
Embodied energy saving (MJ/m^3^)	—	0.7	1.2
Recycled polymer utilized (kg/m^3^)	—	20	40

Note: Assumed emission factor for sand supply (production + typical transport): EF_sand = 10 kg CO_2_/ton.

The simplified indicators were calculated using a screening approach with cradle-to-gate system boundaries and typical emission/energy factors for natural sand production and transport. The CO₂ emission reduction per cubic meter was estimated as:


$$\Delta CO_{\text{2}}=\text{m\textbackslash{}\_substituted}\times \text{EF\textbackslash{}\_sand}$$ (6)

where m_substituted represents the mass of natural sand replaced by polymer shot (20 kg/m^3^ for M5 and 40 kg/m^3^ for M10) and EF_sand is the embodied carbon factor for sand supply (typically = 6–10 kg CO_2_ per ton, i.e., 0.006–0.010 kg CO_2_ per kg, depending on transport distance). A corresponding approach was applied for embodied energy using representative primary energy factors for aggregate supply chains. Because the embodied carbon of natural aggregates is relatively low and the replacement mass is limited, the resulting CO_2_ savings remain small in absolute terms (on the order of 0.1–0.4 kg CO_2_ per m^3^), but become relevant when scaled to large volumes of concrete.

This simplified screening assessment does not represent a full life-cycle assessment and does not include the energy and emissions associated with polymer waste collection and processing, nor potential avoided-burden credits related to polymer waste disposal. These aspects should be addressed in future comprehensive LCA studies.

When considering the embodied carbon footprint of traditional aggregate production, which is estimated to be approximately 6–10 kg CO_2_ per ton, this partial replacement has the potential to reduce overall greenhouse gas emissions, especially when factored in large-scale projects. Specifically, the incorporation of 10% polymer shot by cement mass in concrete results in the diversion of 40 kg of plastic waste per cubic meter, corresponding to the substitution of approximately 40 kg of natural sand per cubic meter. On a scale of 1,000 m^3^ of concrete, this translates to 40 metric tons of waste diverted, a reduction of approximately 400 kg of CO_2_ emissions, and a saving of 1,200 MJ of primary energy associated with aggregate production and transport (see Table 12).

Natural sand is significantly cheaper than recycled polymer streams. Thus, polymer shot is expected to have a higher unit price than virgin sand. However, its practical viability depends on waste valorization and performance benefits (e.g., thermal insulation and slip resistance), which may justify its use in selected applications.

The results demonstrate that even at moderate replacement levels, the use of polymer shot leads to a measurable reduction in the carbon footprint and embodied energy of concrete production. The estimated values are based primarily on the substitution of natural sand. Potential avoided-burden credits associated with polymer waste disposal were not included in this simplified assessment.

Although the current analysis is simplified, it confirms the environmental relevance of the concept and provides a quantitative foundation for future studies including comprehensive life-cycle assessment (LCA), durability testing, and large-scale application of recycled polymer aggregates.

These outcomes emphasize that polymer shot-modified concretes can play a role not only in structural or architectural elements but also in sustainable construction systems combining waste valorization with performance improvement.

All estimated indicators are presented per cubic meter of concrete and correspond to cradle-to-gate system boundaries.

### 4.8. Limitations and future work

Despite the encouraging results, this study has several limitations that should be addressed in future studies to support broader structural and environmental implementation and reliable field performance. First, although the recycled polymer shot was obtained after multi-stage industrial processing and was characterized using particle size distribution, XRF and SEM, additional polymer identification and quality-control methods (e.g., DSC, FTIR or melt flow index) could further strengthen batch-to-batch consistency assessment and standardization. Second, the present work focuses on short-term mechanical, thermal and microstructural performance. Therefore, long-term durability-related properties (e.g., shrinkage, freeze–thaw resistance, carbonation/chloride ingress, sulfate resistance, and aging of the polymer–cement interface) were not investigated and should be addressed in future studies to support broader structural and environmental implementation. In particular, polymers may influence long-term transport properties (permeability and diffusivity), moisture-related behavior (drying/wetting cycles), and time-dependent deformation (shrinkage and creep), as well as the stability of the polymer–cement interfacial transition zone under environmental exposure. Future studies should therefore incorporate durability-focused testing and long-term monitoring to verify whether the microstructural changes observed in the present work translate into improved resistance to environmental degradation and sustained performance over service life.

## 5. Conclusions

This study examined the effect of recycled polymer shot, derived from post-consumer polypropylene waste, on the physical and mechanical performance of concrete. Based on the results obtained, the following conclusions can be drawn:

1. The addition of polymer shot reduced the workability of the mixtures by up to 22%, mainly due to the hydrophobic nature and angular geometry of the polymer particles. Nevertheless, the mixtures retained sufficient flowability for laboratory casting and compaction.
2. The bulk density of the concrete slightly increased with polymer addition (up to 3%), likely due to improved packing density and reduced entrapped air, despite the lower intrinsic density of polymer particles.
3. Thermal conductivity decreased by approximately 40–44%, confirming the potential of polymer shot-modified concrete for applications requiring thermal insulation and energy efficiency.
4. The mechanical properties improved considerably: compressive strength increased by up to 15%, flexural strength by 45%, and tensile strength by 62%, demonstrating a strong reinforcing effect of the polymer shot.
5. The enhanced mechanical behavior can be attributed to improved interfacial bonding, microcrack-bridging, and energy dissipation effects induced by the irregular polymer geometry.
6. The simplified environmental assessment showed measurable benefits, with CO₂ emission reductions of up to 0.4 kg m^−3^ and embodied energy savings of 1.2 MJ m^−3^, highlighting the potential contribution to circular economy objectives.

Overall, the results confirm that recycled polymer shot is a promising additive for producing sustainable, durable, and energy-efficient concretes.

**Practical implications**

The results obtained have direct engineering relevance. The improved slip resistance and strength-to-density ratio indicate potential applications in prefabricated elements, pavements, pedestrian walkways, and thermal insulation layers where both safety and energy performance are critical.

The simplicity of the mix design and the low-cost availability of recycled polymer materials make this approach practical for large-scale implementation without major modifications to standard production procedures.

## Supporting information

S1 File Particle size distribution (PSD) report. This file contains the detailed particle size distribution analysis of the recycled polymer shot used in this study.
(ZIP)

S2 File Raw mechanical and physical test results. This file contains the complete raw dataset underlying the reported mechanical and physical properties, including values used to calculate means, standard deviations, and plots.
(ZIP)
