# Supplementary material for: Recycled polymer shot as sustainable additive for concrete: Mechanical, thermal, and environmental assessment
Source: PLoS One. 2026 Feb 9;21(2):e0342266. doi: 10.1371/journal.pone.0342266 (PMC12885290; doi:10.1371/journal.pone.0342266)
Supplement: S1 File — This file contains the detailed particle size distribution analysis of the recycled polymer shot used in this study. (ZIP) [file pone.0342266.s001.zip › S1 File/S1_File_PSD_report.pdf]

Bettersize

Bettersizer S3 Plus Particle Size Analysis Report

Range : 2um - 3500um

Sample:	S2 KL01	Sample Owner:	Military University Poland
Sample NO.:	2021-3015	Measured By :	3P Instruments
Operator:	FS	Date:	2021-03-30 Time: 15:07:27
Remark:		Size Type:	Equivalent Area Diameter Distribution: Volume

D03= 88.56 um

D06= 117.8 um

D10= 148.6 um

D16= 191.2 um

D25= 254.4 um

D50= 448.5 um

D75= 681.9 um

D84= 787.4 um

D90= 874.5 um

D97= 1025 um

Min Dia.: 16.92 um

Max Dia.: 1473 um

Aver Dia.: 73.91 um

SSA: 8.019m^2/kg

Span: 1.618

Quantity: 299381

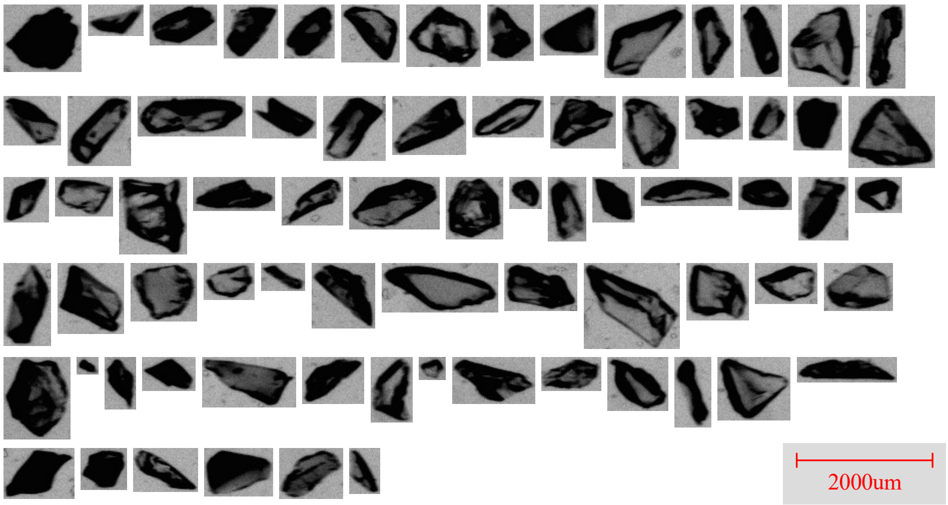

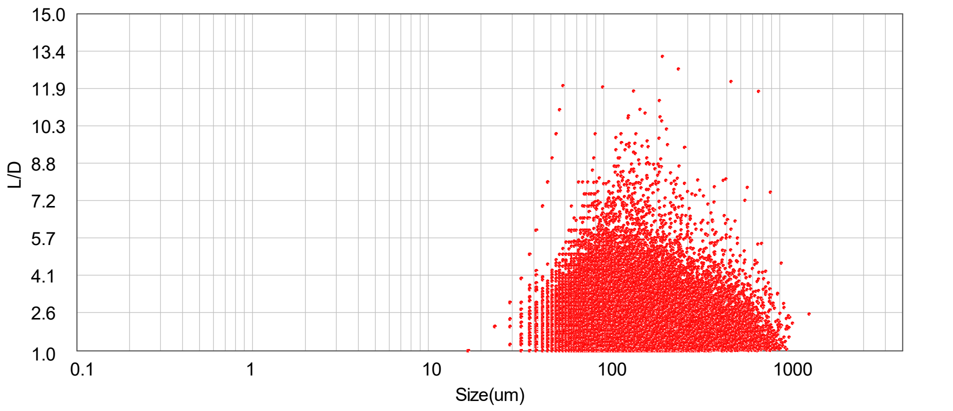

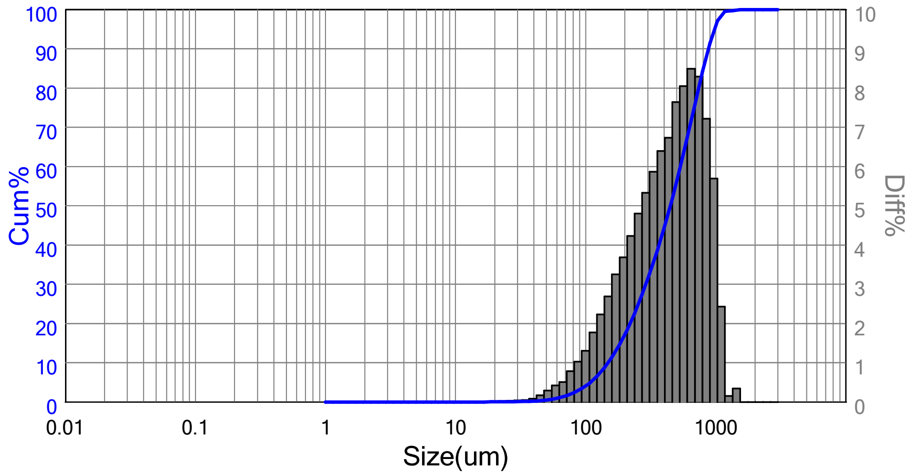

Diam um	Percent
1.000	0.00
2.000	0.00
5.000	0.00
10.00	0.00
20.00	0.01
45.00	0.32
100.0	4.03
200.0	17.22
500.0	55.96
1000	96.18
